# Femtosecond phase-transition in hard x-ray excited bismuth

**DOI:** 10.1038/s41598-018-36216-3

**Published:** 2019-01-24

**Authors:** M. Makita, I. Vartiainen, I. Mohacsi, C. Caleman, A. Diaz, H. O. Jönsson, P. Juranić, N. Medvedev, A. Meents, A. Mozzanica, N. L. Opara, C. Padeste, V. Panneels, V. Saxena, M. Sikorski, S. Song, L. Vera, P. R. Willmott, P. Beaud, C. J. Milne, B. Ziaja-Motyka, C. David

**Affiliations:** 1 Paul Scherrer Institut, CH-5232 Villigen PSI, Switzerland; 2 Synchrotron SOLEIL, L’Orme des Merisiers, 91190 Saint-Aubin, France; 3 CFEL, Deutsches Elektronen-Synchrotron DESY, 22607 Hamburg, Germany; 4 Department of Physics and Astronomy, Uppsala University, SE-751 24 Uppsala, Sweden; 5 Department of Applied physics, KTH Royal Institute of Technology, SE-106 91 Stockholm, Sweden; 6 Institute of Physics, Czech Academy of Sciences, 182 21 Prague 8, Czech Republic; 7 Institute of Plasma Physics, Czech Academy of Sciences, 182 00 Prague 8, Czech Republic; 8 C-CINA Biozentrum, University of Basel, CH-4058 Basel, Switzerland; 9 Institute for Plasma Research, Bhat Gandhinagar, 382428 India; 10 Linac Coherent Light Source, SLAC National Accelerator Laboratory, Menlo Park, California, 94025 USA; 11 Institute of Nuclear Physics, Polish Academy of Sciences, 31-342 Krakow, Poland

## Abstract

The evolution of bismuth crystal structure upon excitation of its A_1g_ phonon has been intensely studied with short pulse optical lasers. Here we present the first-time observation of a hard x-ray induced ultrafast phase transition in a bismuth single crystal at high intensities (~10^14^ W/cm^2^). The lattice evolution was followed using a recently demonstrated x-ray single-shot probing setup. The time evolution of the (111) Bragg peak intensity showed strong dependence on the excitation fluence. After exposure to a sufficiently intense x-ray pulse, the peak intensity dropped to zero within 300 fs, i.e. faster than one oscillation period of the A_1g_ mode at room temperature. Our analysis indicates a nonthermal origin of a lattice disordering process, and excludes interpretations based on electron-ion equilibration process, or on thermodynamic heating process leading to plasma formation.

## Introduction

The typical response time of the internal microscopic degrees of freedom in a solid, such as the arrangement of the electrons and atoms, ranges between few fs to few ps. Ultrashort laser pulses can excite materials on time scales faster than those response times, often revealing unique behaviour^1–5^ and furthering the understanding of the interactions between electrons and atomic lattice^6–8^. In the case of ultrafast melting, photoexcitation drives the material in a highly non-equilibrium state where the electrons are excited while the lattice is still cold. As the electrons thermalize, the lattice disorders in hundreds of femtoseconds due to a significant shift in the atomic potential energy surface (PES) driven by the excited electrons. Bismuth (Bi), well known for its Peierls distorted lattice structure, is an important example of such a phase transition.

The crystal lattice of Bi can be readily excited by ultrashort laser pulses promoting electronic excitations that typically trigger a coherent oscillation of the optical Γ-point A_1g_ phonon mode^1–4,9^. The characteristic parameters of this oscillation, for example its frequency (~2.9 THz^10,11^), are strongly dependent on the excitation intensity and are directly correlated with the out-of-equilibrium potential energy surface^12^ (PES). Results from a pioneering infrared-pump experiment^13^ and subsequently from theoretical models^8^, suggested presence of a non-thermal melting process in Bi at an absorbed dose above its thermal melting threshold. To date, however, to the best of our knowledge, neither experiments nor calculations have been able to describe the dynamics in a range above an absorbed dose of 1.2 eV/atom^13^, which is still well below a strong fast ionisation regime. In the interest of time-resolved studies, several experiments explored excitation regimes up to the regime of nonthermal melting in Bi^3,9,14^ and in other materials with state-of-the-art techniques^15,16^. Up to now, structural studies at high temporal resolution (<100 fs) sensitive to the excitation of phonon modes in Bi have never been carried out neither in the non-thermal melting regime or above it. It is therefore of a fundamental interest to investigate the time-resolved dynamical lattice response of Bi to an intense electronic excitations, with high temporal resolution.

To address this question, we performed a study of the ultrafast lattice dynamics of a bismuth bulk crystal, excited with a femtosecond hard x-ray pulse. The advantages of using hard x-rays, instead of optical lasers, are: (i) x-rays allow to highly excite the material with negligible non-linear effects from electric and magnetic fields of the focused laser^17^. In this way a purely electronic response of the material can be studied, and (ii) x-ray irradiation creates a low photoelectron density gradient within the pump pulse penetration depth under an incidence close to normal, yielding a relatively homogeneous secondary electron density therein. The technical difficulties typically associated with an x-ray pump and x-ray probe experiment using SASE pulses^18^ were overcome by using a novel x-ray splitting setup^19^. As a result, an extremely high temporal resolution was achieved, limited only by the XFEL pulse duration. Importantly, this technique allows for combined characteristics which: (1) is free of any timing and spatial jitter between the pump and the probe, or between the probe beams, (2) provides consecutive probing (8 in this work) from a single x-ray pulse, thus eliminating stochastic effects, and (3) allows for independent focusing between pump and the probe pulses.

## Results

**Figure 1:**
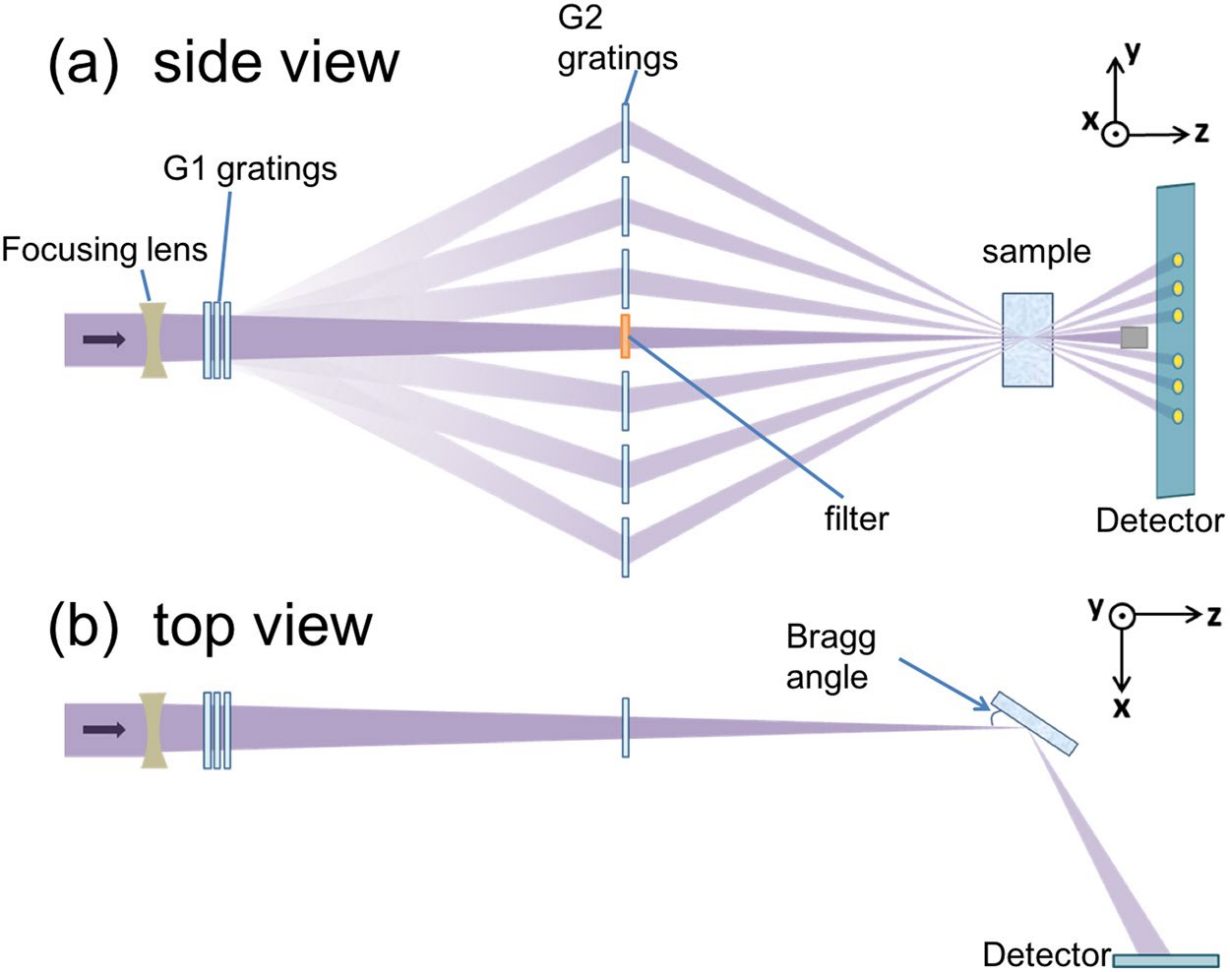
(**a**) Y-Z plane view of the setup. The label ‘G1 gratings’ denotes a stack of 10 different diamond gratings. Only three are shown here for simplicity. Each of these gratings diffracts a small portion of the incoming x-ray pulse in the y-z plane, at varying angles defined by their pitch. The diffracted pulses are then re-diffracted back by the G2-gratings, to overlap with the transmitted primary pulse within ±3 μm precision. (**b**) X-Z plane view of the setup. All the transmitted and diffracted pulses satisfy the Bragg condition of the sample in the x-z plane. The pulses that diffract from the sample are separated along the y-axis and aligned along the x-axis. They are then recorded on a 2D detector (JUNGFRAU).

The experiment was performed at the XCS station^20^ of the Linac Coherent Light Source (LCLS)^21^, using an x-ray photon energy of 5 keV, a nominal pulse energy of 2 mJ and a nominal pulse duration of 35 fs FWHM. The schematic arrangement of the transmission gratings is illustrated in Fig. 1a. The transmitted part of the x-ray pulse through the gratings acted as a ‘pump’, whilst those diffracted by the gratings ‘probed’ the sample at precise time delays determined by their extended optical paths relative to the transmitted pulse. On the sample, half of the probe beams are spatially overlapped with the pump pulse, while the other half was separated by 70 μm from the pumped region. The uniqueness of the setup has been demonstrated previously^19^. The two major differences from the previous work in this experiment are: finer time steps of 20–50 fs for an overall coverage of 300 fs, allowing higher probing time resolution, and, 2–5 times higher grating diffraction efficiency resulting in better signal-to-noise ratio. Further details about the setup can be found in the Methods and in refs.^19,22^. To ensure the spatial overlap, spot sizes of the pump and the probe pulses were focused to a FWHM of 35 ± 5 μm and 12.5 ± 2.5 μm, respectively. The sample was oriented so as to direct the Bragg reflections of all the pulses onto a 2D detector.

**Figure 2:**
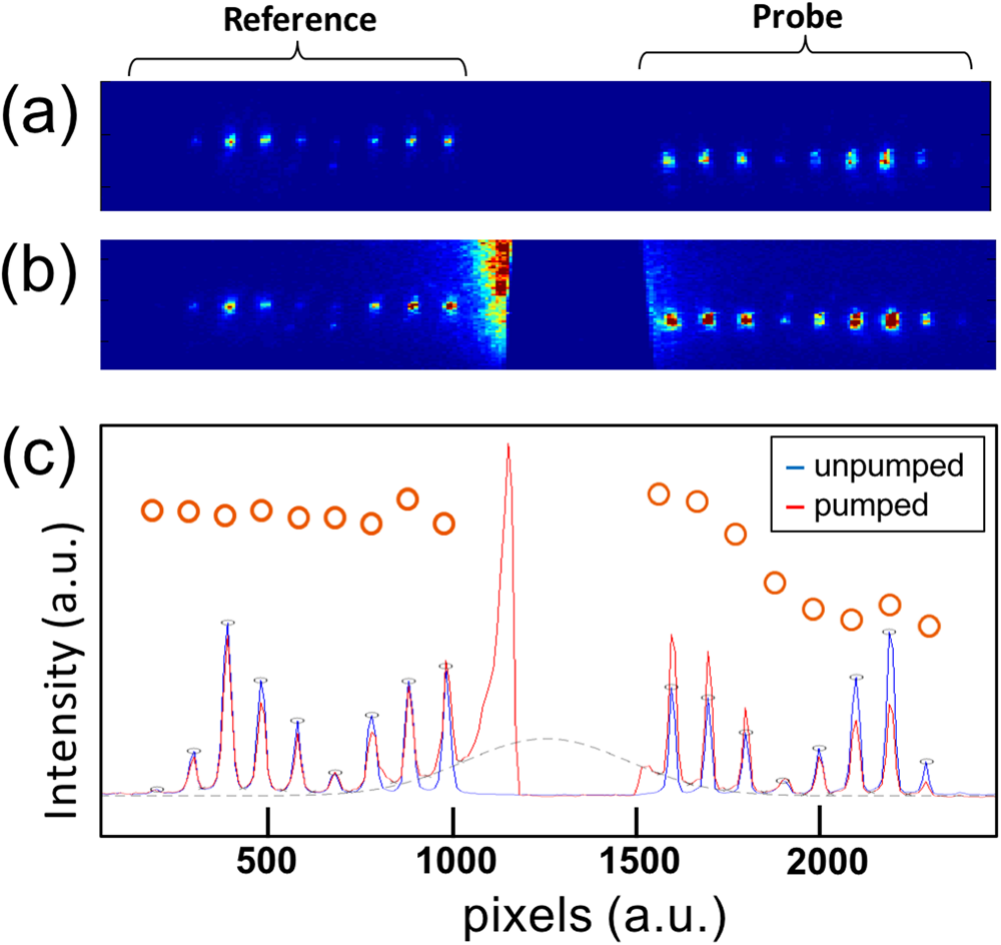
(**a**,**b**) Typical raw images showing (**a**) unpumped, and (**b**) pumped events of Bragg reflection signals. The pump pulse is blocked in front of the detector. The integrated plots of these images are shown in (**c**), red (pumped) and blue (unpumped) lines. The time delay increases symmetrically left and right from the pump-pulse at the centre. A dashed grey line indicates the 1D Gaussian background level in the pumped case. After background removal, the pumped signals are then integrated and normalised by their counterpart from unpumped signals. The normalised signals are plotted as red open circles.

Figure 2a,b show typical single-shot images of the Bi (111) Bragg peak reflections which is sensitive to the A_1g_ phonon mode, as recorded on the detector. Diffraction patterns with and without the pump pulse are compared (Fig. 2a,b). For both images, the group of signals on the left-hand side, marked as “reference”, probed the unpumped region of the sample, while that marked as “probe” on the right probed the excited area of the sample. The delay time increases as the signal distance increase from the (blocked) pump pulse at the centre of the image. The flare caused by the pump beam was fitted with a 1D Gaussian profile for background removal (Fig. 2c).

**Figure 3:**
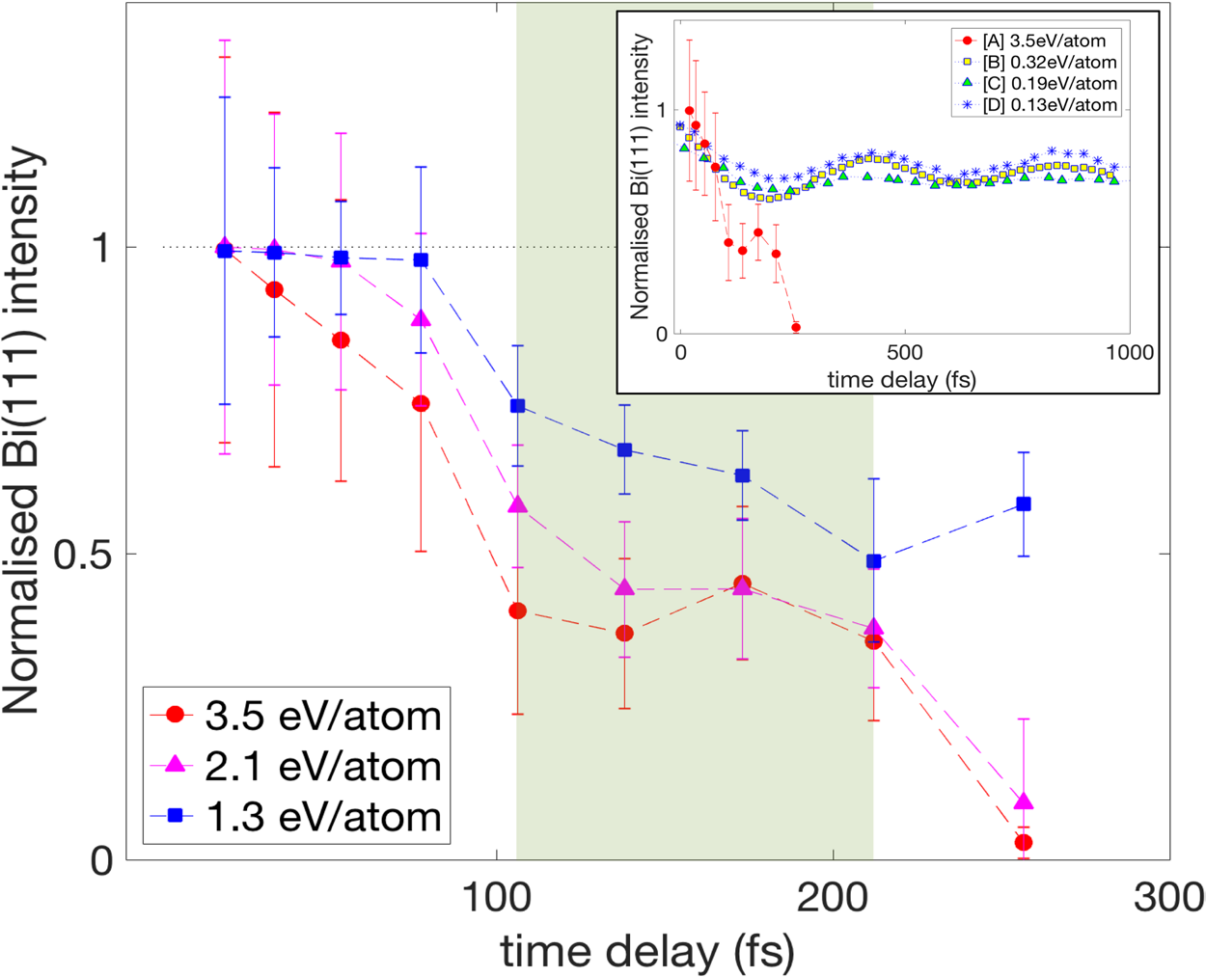
Temporal evolution of the normalised signals from x-ray irradiated Bi crystal at three different absorbed doses: 3.5 eV/atom (red circles), 2.1 eV/atom (magenta triangles) and 1.3 eV/atom (blue squares). Error bars are taken from standard deviation. Dashed lines are guides for the eye. The green shaded area marks roughly the plateau region discussed in the text. The inset graph shows the comparison of the case with [A] 3.5 eV/atom absorbed dose from 5 keV x-ray pulse, with the extrapolated data from previous reports on optical excitation of Bi at various absorbed doses: [B] Harmand *et al*. ([9]), [C] Fritz *et al*. ([2]), and [D] Johnson *et al*. ([3]).

Figure 3 shows the time evolution of the (111) reflection recorded for different x-ray pump fluences, with the relative pump incidence at time 0 (centre of the pump pulse). The fluence levels are 2.46 J/cm^2^, 1.48 J/cm^2^, and 0.91 J/cm^2^, corresponding to the average absorbed dose values of 3.5 eV/atom, 2.1 eV/atom, and 1.3 eV/atom, respectively. (The chosen x-ray energy does not induce a resonant absorption by inner-shell electrons). Prior to the pump events, probe pulse intensities were measured and confirmed low enough not to damage or affect the crystal. On average, 50 ± 10 selected shots for each fluence level were used. We emphasize here that the signal dynamics seen in Fig. 3 cannot be due to x-ray intensity and spectral fluctuations, or due to any crystal imperfections, since the time sequences were taken from the same pulse in single shot and from the same region of the crystal.

The dynamics at the lowest absorption dose of 1.3 eV/atom (at 0.91 J/cm^2^) in our case could be considered similar to the highest dose cases presented in ref.^13^ (1.2 eV/atom for Bi) and in ref.^23^ (0.35 eV/atom for Sb) – implying that the PES map at this x-ray dose should be highly anharmonic and described by a single-minimum-well. For such a case, it is expected that the disordering of the lattice follows a relatively direct path, possibly by a direct anharmonic coupling of the initially excited A_1g_ motion coordinate to that of the degenerate E_g_ optical mode^8,13^.

For the absorbed doses of 2.1 eV/atom and 3.5 eV/atom, the intensity drops to zero within ~300 fs, faster than one oscillation period of the unperturbed Bi A_1g_ phonon mode (342 fs, 2.92 THz)^10–12^. This is also highlighted in the inset graph in Fig. 3, which compares our observation to previous Bi (111) diffraction experiments in which bond softening or lattice damage at later times were observed^2,3,9^. Our signal decays in less than half a picosecond ruling out the possibility of a thermal melting process. We also note that the observed femtosecond transition triggered by 10^13^~10^14^ W/cm^2^ pump intensity excludes the possibility of fast collisional heating through photoexcited electrons^24^ as a dominant heating process. More details about these analyses will be given in the discussion below.

An intermediate signal plateau present in all fluence cases shown in Fig. 3 (shaded green area) has been observed at a comparable timescale in x-ray irradiated diamond within its transient transmission of optical pulses^25^. An exponential fitting to extrapolate the decay time constant is possible for reflections from lattice insensitive to optical phonon-modes^5,13^. In our case, however, due to the sensitivity of (111) reflection to the A_1g_ phonon mode, we cannot uniquely exclude the possible origin of the plateau in Fig. 3 from a damped oscillatory motion along the distortion coordinate^26,27^. In such a case, the major initial atomic potential shift would be along the (111) direction, leading to minima of diffracted intensity when Bi atomic potential passes through or near the lower symmetry equilibrium position of the parent cubic lattice. A possibility of such a process could be validated in the future by performing dedicated experiments with a longer time delay window.

## Discussion

In the thermal melting regime, dramatic changes in the crystal structure can only be observed after significant atomic heating due to the electron-ion energy exchange. This process takes time typically of the order of ps^28,29^ at the pump pulse fluences considered here. Therefore, the complete signal drop within 300 fs, seen in Fig. 3, casts out the lattice disorder through its thermalisation as a possible cause.

If, on the other hand, the diffraction signal drop is due to strong ionisation only, one would expect a significant change of the Bi atomic scattering factor due to ionisation of core-electrons. However an ionisation degree estimated with the non-local thermodynamic-equilibrium (non-LTE) radiation transfer code, CRETIN^30^, points to less than 1 electron per atom being excited in the first 100 fs after the pump, at our highest intensity of 10^14^ W/cm^2^ (equivalent to 3.5 eV/atom) (see Supplementary Fig. (ii)). Indeed, an estimate of the scattered intensity based on XATOM code^31^ suggest that, to realise the observed signal drop, the normalised scattering factors of Bi should be at least ~0.8. This corresponds to an average ionisation degree of more than ~10 per atom in the initial state (see Supplementary Fig. (i)). To reach such a degree of ionisation would require orders of magnitude higher x-ray intensity than we have in this experiment, which would push the sample into a plasma state.

This leaves a non-thermal melting process as the most likely interpretation of the observed transition, where a modification of interatomic potential due to high electronic excitation induces a lattice instability^13,32^. Given the average absorbed pump doses (1.3~3.5 eV/atom) in our cases, we conclude that 0.3~1.4 atoms for every 1000 atoms underwent photoabsorption. Therefore, ~0.1% of the atoms have one inner-shell hole due to photo-excitation, which is immediately filled through an Auger process occurring in a sub-fs timescale^31^. Immediately after photo-excitation, this process creates only ~0.1% holes in the valence or conduction bands, and the core holes are promptly filled. We can then assume that in the first instants after photoexcitation the interatomic potential is not significantly perturbed. Meanwhile the free energy of the sample increases by the amount of the absorbed energy, which is stored in high energy (>keV) photoelectrons. In a conventional non-thermal melting processes, the following dynamics has been discussed, especially for the case of semiconductors^5,32,33^. A fast electron distributes its energy through collisional interactions repetitively until it settles down into bound states. At the same time the electron cascading processes further excite electrons from the valence to the conduction band, altering the electron density distribution and thus the interatomic potential, and consequently leading to lattice disorder.

Given that x-ray diffraction is mainly sensitive to the changes in core electron density, it should be noted that Bragg diffraction signal is, in principle, not directly sensitive to the PES. Indeed, for hard x-ray excited Si with similar absorbed doses to our case^5^, the start of the Bragg diffraction signal decay was observed after ~110 fs from the incidence of the pump. The similar excitation condition in Bi in our case also implies a significantly low ionisation degree, thus low photoelectron population, despite the absorbed dose is up to a few times higher than its damage threshold^13^. For reflections insensitive to optical modes, similarly to the Si or diamond cases^5,25,33^, one could expect a short period of delay until the onset of signal drop can be observed. However, from Bi (111) the onset of the signal decay is almost immediate, especially for the highest excitation case. It indicates an atomic dislocation process occurring in parallel to non-thermal melting process.

The fluence dependence of our measurements (as seen in Fig. 3), carries additional information on the possible phenomena occurring upon photoexcitation. Scenarios such as thermal excitation of the lattice (Debye-Waller) would exhibit a fluence dependence only in the amplitude of the exponentially decreasing diffracted intensity. In contrast, here both the amplitude and the gradient leading to the normalised intensity of ~0.5 in Fig. 3 show fluence dependence – an observation compatible with the displacive excitation of coherent phonons (DECP)^12^. Theoretical modelling of the DECP process is primarily dependent on the pump pulse duration (shorter than the phonon oscillation period) and on the absorbed power density. Presence of energetic excited electrons leads to excitation of many outer electrons, thus a coherent motion of the lattice is induced as a result of changes of PES, likely contributing to the building up of a non-thermal melting process.

Assuming that DECP process is initiated, the potential implication following here is a strong anharmonic coupling between the A_1g_ and the E_g_ optical modes leading to a phase transition on the timescale less than one period of the A_1g_ mode, as discussed also in ref.^8^. We therefore speculate that this coherent lattice motion could be the precursor to the observed non-thermal melting process on such a rapid timescale. These stages of transition dynamics initiated by hard x-rays are thus most likely relevant for materials with *A*_1_ optical modes in general.

In conclusion, we have performed the first-time observation of x-ray induced lattice disordering in Bi occurring within less than 300 fs. The (111) Bragg peak dynamics indicates that the phase transition proceeds as a complex multistep process. Our data set an important benchmark for future experiments and modelling of the hard x-ray induced ultrafast phase-transition in Bi, which should reproduce the observed fluence dependence. For that purpose, there is a strong need for a development of dedicated theoretical models capable of following such complex non-equilibrium dynamics in time. Promising candidates currently being developed are time-dependent Density Functional Theory or Hartree-Fock based schemes.

## Methods

### Experimental setup

The schematic arrangement of the transmission gratings is illustrated in Fig. 1a. A set of 10 linear gratings (G1 gratings), made of diamond, was placed 0.3 m downstream from the beamline exit window. A set of 20 linear gratings (G2 gratings), made of Ir and SiO_2_, was positioned 3.3 m downstream of the G1 gratings to receive the diffracted beams from the G1 gratings. All the gratings were fabricated by means of electron-lithography and reactive ion etching, at the Laboratory for Micro- and Nanotechnology, Paul Scherrer Institut. The sample was positioned 6.6 m downstream of the G1 gratings, accepting both the transmitted (pump) beam and 20 diffracted (probe) beams from the G2 gratings. On the sample, half of the probe beams spatially overlap with the pump pulse, while the other half is separated by 70 μm to the unpumped region. In this way, the whole sequence of pumped and unpumped signals are obtained simultaneously, from a single x-ray pulse. In this experiment, the grating efficiencies are enhanced by factors of 2~5 compared to those reported earlier. The grating pitches were optimized to sequentially increase the delay timings with precisely defined intervals of 20 fs to 50 fs. This small time steps were continued up to ~300 fs delay, with the probe focus on early-onset of the damage to the crystal.

The Bragg angle for the (111) reflection in Bi at 5 keV is 18° with a measured rocking curve width of 0.2°. The varying incident angles in y-z plane of the pump and probe beams do not affect the Bragg condition, because the diffraction angle from the sample is only sensitive to the angle in the x-z plane, which is perpendicular to the gratings diffraction plane (Fig. 1b). Each pulse are then incident at the detector at spatially separated locations due to the angular separations in the y-z planes created by the diffraction gratings, thus retaining the delay-time information. Fresh sample surface was used for every shot.

The Bragg reflected probe beam signals were recorded using the JUNGFRAU detector, an integrating two-dimensional pixel detector comprised of arrays of 75 × 75 μm^2^ pixels, with single photon sensitivity over a dynamic range of 2.5 × 10^4^ for 5 keV photons. More details about the JUNGFRAU detector can be found in^34^. The intensity of each diffracted peak on the detector was determined by integrating the counts up to half the distance to the neighbouring peaks. The integrated peak intensities were then normalised against the unpumped counterparts, which were then rescaled using the normalised and averaged “reference” signals.

The size of the pump beam spot was determined by observing the attenuated x-ray beam on a YAG screen at the sample position, using a microscope lens and a Charge Coupled Device (CCD) detector. The different spot sizes of the pump and the probe pulses were achieved by using Be focusing lenses positioned at two places. The first set was placed upstream of the G1 gratings and therefore affected the focusing of the direct pump beam and the probe beams. The other set was placed on the optical axis at the G2 grating position (not shown in the figure) and only affected the focusing of the pump pulse. This arrangement allowed us to choose the size of the pump and the probe beams independently.

The unattenuated pump pulse energy at the sample position (after passing through the gratings and the focusing lenses) was measured with a calorimeter to be ~100 μJ, which is ~5% of the nominal total emitted pulse energy. In order to vary the fluence level, but not the probe intensities, the pump pulse was attenuated using 10 μm and 20 μm Al foils placed on the optical axis of the transmitted beam, close to the G2 grating (Fig. 1a). With these conditions, the pump beam fluence varied from 0.9 to 2.5 J/cm^2^. The energy of each probe beam was 0.01 to 0.2% of the pump beam at the sample position. In the absence of the pump beam, the crystal structure remains intact, confirming that the probe pulses are sufficiently weak to not damage the crystal.

## Electronic supplementary material

Supplementary information

## References

[1] Sokolowski-Tinten K (2003). Femtosecond X-ray measurement of coherent lattice vibrations near the Lindemann stability limit. Nature.

[2] Fritz DM (2007). Ultrafast Bond Softening in Bismuth: Science.

[3] Johnson SL (2009). Directly Observing Squeezed Phonon States with Femtosecond X-ray Diffraction. Phys. Rev. Lett..

[4] Johnson SL (2008). Nanoscale Depth-Resolved Coherent Femtosecond Motion in Laser-Excited Bismuth. Phys. Rev. Lett..

[5] Pardini T (2018). Delayed Onset of Nonthermal Melting in Single-Crystal Silicon Pumped with Hard X Rays. Phys. Rev. Lett..

[6] Murray ÉD, Fahy S (2015). First-principles calculation of femtosecond symmetry-breaking atomic forces in photoexcited bismuth. Phys. Rev. Lett..

[7] Fahy S, Murray ÉD, Reis DA (2016). Resonant squeezing and the anharmonic decay of coherent phonons. Phys. Rev. B.

[8] Zijlstra ES, Tatarinova LL, Garcia ME (2006). Laser-induced phonon-phonon interactions in bismuth. Phys. Rev. B.

[9] Harmand M (2013). Achieving few-femtosecond time-sorting at hard X-ray free-electron lasers. Nat. Photonics.

[10] Fischer P, Sosnowska I, Szymanski M (1977). Debye-Waller factor and thermal expansion of arsenic, antimony and bismuth. J. Phys. C Solid State Phys..

[11] Gonze X, Michenaud J-P, Vigneron J-P (1990). First-principles study of As, Sb, and Bi electronic properties. Phys. Rev. B.

[12] Zeiger HJ (1992). Theory for displacive excitation of coherent phonons. Phys. Rev. B.

[13] Sciaini G (2009). Electronic acceleration of atomic motions and disordering in bismuth. Nature.

[14] Lindenberg AM (2005). Atomic-Scale Visualization of Inertial Dynamics. Science.

[15] Musumeci P, Cesar D, Maxson J (2017). Double-shot MeV electron diffraction and microscopy. Struct. Dyn..

[16] Otto, M. R., Renée de Cotret, L. P., Stern, M. J. & Siwick, B. J. Solving the jitter problem in microwave compressed ultrafast electron diffraction instruments: Robust sub-50 fs cavity-laser phase stabilization. *Struct*. *Dyn*. **4,** 051101 (2017). 10.1063/1.4989960 PMC5552402 28852686

[17] Gibbon P, Förster E (1996). Short-pulse laser - plasma interactions. Plasma Phys. Control. Fusion.

[18] Emma P (2004). Femtosecond and subfemtosecond x-ray pulses from a self-amplified spontaneous-emission-based free-electron laser. Phys. Rev. Lett..

[19] David C (2015). Following the dynamics of matter with femtosecond precision using the X-ray streaking method. Sci. Rep..

[20] Alonso-Mori R (2015). The X-ray Correlation Spectroscopy instrument at the Linac Coherent Light Source. J. Synchrotron Radiat..

[21] Emma P (2010). First lasing and operation of an ångstrom-wavelength free-electron laser. Nat. Photonics.

[22] Opara NL (2018). Demonstration of femtosecond X-ray pump X-ray probe diffraction on protein crystals. Struct. Dyn..

[23] Bauerhenne B, Zijlstra ES, Garcia ME (2017). Molecular dynamics simulations of a femtosecond-laser-induced solid-to-solid transition in antimony. Appl. Phys. A.

[24] Vinko SM (2012). Creation and diagnosis of a solid-density plasma with an X-ray free-electron laser. Nature.

[25] Tavella F (2017). Soft x-ray induced femtosecond solid-to-solid phase transition. High Energy Density Phys..

[26] Beaud P (2014). A time-dependent order parameter for ultrafast photoinduced phase transitions. Nat. Mater..

[27] Huber T (2014). Coherent structural dynamics of a prototypical charge-density-wave-to-metal transition. Phys. Rev. Lett..

[28] Sokolowski-Tinten K (2015). Thickness-dependent electron – lattice equilibration in laser-excited thin bismuth films. New J. Phys..

[29] Arnaud B, Giret Y (2013). Electron cooling and Debye-Waller effect in photoexcited bismuth. Phys. Rev. Lett..

[30] Scott HA (2001). Cretin—a radiative transfer capability for laboratory plasmas. J. Quant. Spectrosc. Radiat. Transf..

[31] Son S-K, Young L, Santra R (2011). Impact of hollow-atom formation on coherent x-ray scattering at high intensity. Phys. Rev. A.

[32] Medvedev N, Jeschke HO, Ziaja B (2013). Nonthermal phase transitions in semiconductors induced by a femtosecond extreme ultraviolet laser pulse. New J. Phys..

[33] Medvedev N, Li Z, Ziaja B (2015). Thermal and nonthermal melting of silicon under femtosecond x-ray irradiation. Phys. Rev. B.

[34] Mozzanica A (2016). Characterization results of the JUNGFRAU full scale readout ASIC. J. Instrum..

