# Supplementary material for: Femtosecond phase-transition in hard x-ray excited bismuth
Source: Sci Rep. 2019 Jan 24;9:602. doi: 10.1038/s41598-018-36216-3 (PMC6345934; doi:10.1038/s41598-018-36216-3)
Supplement: Supplementary file 1 — Supplementary information [file 41598_2018_36216_MOESM1_ESM.pdf]

# Femtosecond phase-transition in hard x-ray excited bismuth

*M. Makita [a], I. Vartiainen [a], I. Mohacsi [a,b], C. Caleman [c,d], A. Diaz [a], H. O. Jönsson [d, e], P. Juranić [a], N. Medvedev [f, g], A. Meents [c], A. Mozzanica [a], N. L. Opara [a, h], C. Padeste [a], V. Panneels [a], V. Saxena [c, i], M. Sikorski [j], S. Song [j], L. Vera [a], P. R. Willmott [a], P. Beaud [a], C.J. Milne [a], B. Ziaja-Motyka [c, k], and C. David [a]

*(a) Paul Scherrer Institut, CH-5232, Villigen PSI, Switzerland*
*(b) Synchrotron SOLEIL, L'Orme des Merisiers, 91190 Saint-Aubin, France*
*(c) CFEL, Deutsches Elektronen-Synchrotron DESY, 22607 Hamburg, Germany*
*(d) Department of Physics and Astronomy, Uppsala University, SE-751 24 Uppsala, Sweden*
*(e) Department of Applied Physics, KTH Royal Institute of Technology, SE-106 91 Stockholm, Sweden*
*(f) Institute of Physics, Czech Academy of Sciences, 182 21 Prague 8, Czech Republic*
*(g) Institute of Plasma Physics, Czech Academy of Sciences, 182 00 Prague 8, Czech Republic*
*(h) C-CINA Biozentrum, University of Basel, CH-4058, Switzerland*
*(i) Institute for Plasma Research, Bhat, Gandhinagar 382428, India*
*(j) Linac Coherent Light Source, SLAC National Accelerator Laboratory, Menlo Park, California, 94025, USA*
*(k) Institute of Nuclear Physics, Polish Academy of Sciences, 31-342 Krakow, Poland*

**Corresponding author email address: mikako.makita@xfel.eu*

## Supplementary Information

### Table of Contents:

1) **Scattering factor and ionisation degree estimate with XATOM**
2) **Ionisation degree calculation with CRETIN**

### 1) Scattering factor and ionisation degree estimate with XATOM

Assuming that the diffracted Bragg signal scales with the scattering factor, the expected average photoionization degree of atoms within the x-ray irradiated Bi bulk is estimated with XATOM code[1].

The x-ray excited Bi was treated iteratively (up to ~100 iterations for convergence), for each charge state of Bi, in a spherical coordinate system. For each iteration, effective single-electron Schroedinger equation is solved within Hartree-Fock-Slater (HFS) model, with Latter's correction in the asymptotic behaviour of the HFS potential. To calculate atomic data for all individual electronic configurations, called Self Consistent Field (SCF) procedure, an energy error threshold of 1e-8 atomic units (a.u.), was used. This error includes multiple-hole states of arbitrary atomic species, as well as the cross sections and rates of x-ray-induced atomic processes. For this calculation, we have used approximately 200 grid points in the radial direction (more than 0-50 a.u. radius) and about 100 grid points in the angular direction (over 0-20 a.u. momentum).

Figure (i) shows the atomic scattering factors for different charge states of Bi, as the functions of momentum transfer. Based on this result, the required degree of ionisation in the initial state, to reach the observed intensity drop, is estimated to be at least 10 - which is contradictory to the low pump intensity, and thus photoabsorption probabilities corresponding to the fluence used in the experiment.

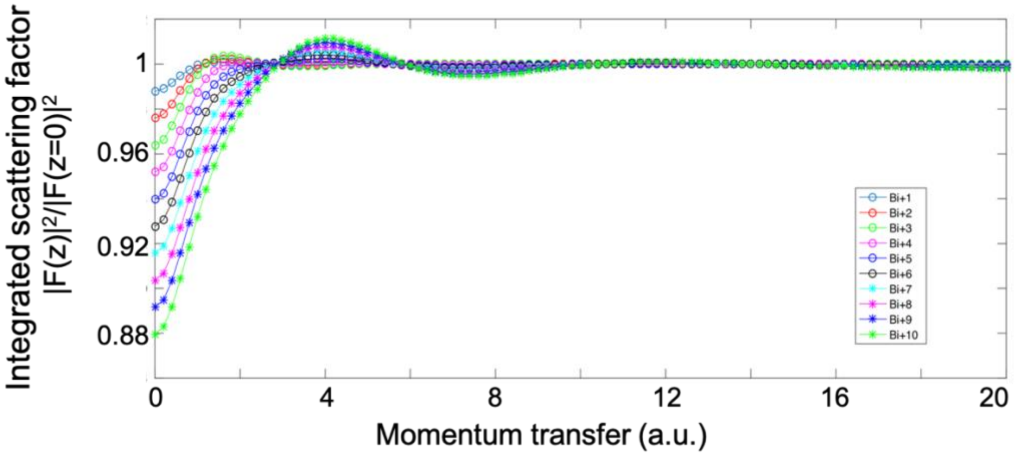


Figure (i): The scattering factors (y-axis) for various Bi ions. The y-axis is the integrated scattering factor (ISF) for different charge states, normalised by the ISF for neutral Bi. The x-axis is the momentum transfer in a.u.. The plot suggests that the average ionisation degree in Bi bulk would have to increase significantly within the 300 fs timescale, in order to explain the observed Bragg peak decay seen in Fig. 3 of the main manuscript as due to the decrease in the scattering factor.

**2) Ionisation degree calculation with CRETIN:**

Ionisation degree of Bi under 5 keV x-ray excitation was estimated using the non-local thermodynamic equilibrium (non-LTE) plasma simulation code CRETIN[2]. For the experiment condition presented in the manuscript, the plasma model is expected to provide an overview description of the material under x-ray excitation, such as the ionisation state, or electron temperature, as a function of time (more examples could be found in refs. [3,4]).

The simulation parameters are matched with the fluence and photon energy in the experiment, i.e. photon energy of 5 keV, pulse duration of 35 fs with a flat-top X-ray pulse model. The structure is not explicitly modelled in this code and the material is treated as a continuum. Instead, the sample is simulated in nanometre-sized zones, where continuum lowering governed by Stewart-Pyatt degeneracy is applied to compensate for the lowering of ionisation potentials. The code allows electron and ion temperatures to be different but assume a Maxwellian distribution of kinetic energies within those populations. Using this concept, rate equations are solved to calculate the time evolution of the system with changing population

of electronic states, radiation transport including energy dependent absorption and opacity changes.

The calculation keeps track of the average ionisation and temperatures of electrons and ions separately, while excluding any local structural information assuming that the local density of the atoms is homogeneous within the simulation duration (~100 fs). It was found that even at the highest fluence case, the average ionisation degree per atom was around 0.8 after 100 fs, where the primary modes of ionisation were direct photoionisation and Auger ionisation. It also suggests that many of the capabilities of the code related to highly ionized plasma states were not activated even for the highest dose case.

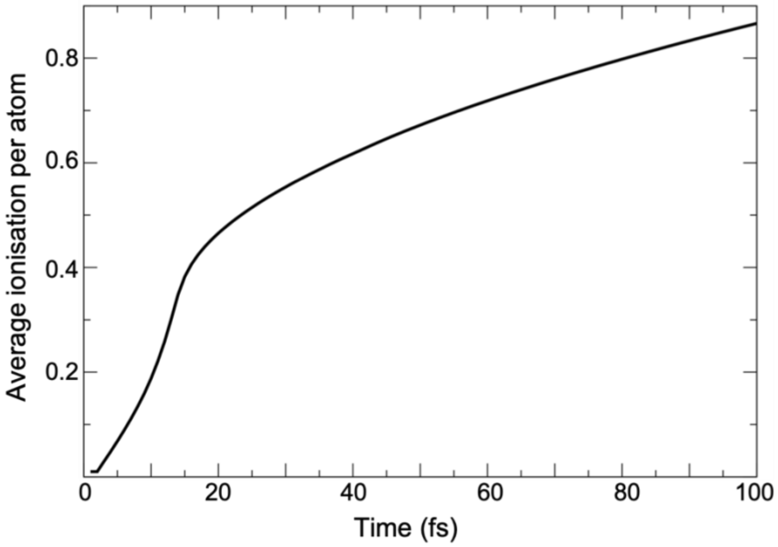


Figure (ii): Average ionisation degree per atom of bismuth for the highest fluence case (3.5 eV/atom). Time zero denotes the pump pulse incidence. The average ionisation degree is less than 1, at 100 fs after the laser incidence, indicating that the sample to reach plasma state on the timescale of < 300 fs is unlikely.


1. Son, S.-K., Young, L. & Santra, R. Impact of hollow-atom formation on coherent x-ray scattering at high intensity. *Phys. Rev. A* **83,** 033402 (2011).
2. Scott, H. A. Cretin—a radiative transfer capability for laboratory plasmas. *J. Quant. Spectrosc. Radiat. Transf.* **71,** 689–701 (2001).
3. Beyerlein, K. R. *et al.* Ultrafast nonthermal heating of water initiated by an X-ray Free-Electron Laser. *Proc. Natl. Acad. Sci. U. S. A.* **115,** 5652–5657 (2018).
4. Nass, K. *et al.* Indications of radiation damage in ferredoxin microcrystals using high-intensity X-FEL beams. *J. Synchrotron Radiat.* **22,** 225–238 (2015).
